# Image‐Derived Volumetry During Forced Expiration Using a 3D Fermat Looped, Orthogonally Encoded Trajectory

**DOI:** 10.1002/mrm.70545

**Published:** 2026-07-27

**Authors:** Sebastian Scheidel, Viktor Hartung, Matthias Stephan Anders, Corona Metz, Herbert Köstler, Simon Veldhoen, Tobias Wech

**Affiliations:** ^1^ Department of Diagnostic and Interventional Radiology University Hospital Würzburg Würzburg Germany; ^2^ Department of Pediatric Radiology Charité‐Universitätsmedizin Berlin, Corporate Member of Freie Universität Berlin and Humboldt‐Universität zu Berlin Berlin Germany

**Keywords:** compressed sensing, forced expiration, lung imaging, spirometry

## Abstract

**Purpose:**

To enable MRI‐based nongated 3D depiction of forced expiration with both high spatio‐temporal resolution and fidelity of lung dynamics.

**Methods:**

An undersampled 3D Fermat looped, orthogonally encoded trajectory (FLORET) was optimized for nonsegmented dynamic acquisitions during forced expiration and a corresponding model‐based reconstruction pipeline promoting spatial and temporal total variation was implemented. The approach was first optimized and validated using a lung phantom, specifically constructed for this study and was then translated to in vivo measurements. This allowed high temporal resolution of 200 ms and isotropic spatial resolution of 5 mm.

**Results:**

Volumes derived by segmenting the MR images of the phantom demonstrated excellent agreement with the air exchange measured directly at the phantom within the bore, therefore confirming the validity of the approach. In both phantom and in vivo experiments, the dynamic deflation process could be successfully depicted, confirming the potential for assessing forced expiration with 3D UTE FLORET MRI. From the segmented volumes in vivo, quantitative parameters were derived, including the duration of the dynamic phase (2.8 ± 0.2 s), the time to 90% deflation (1.8 ± 0.2 s), and the volume change within the first second (2.8 ± 0.2 L).

**Conclusion:**

The phantom and in vivo studies demonstrate, that the 3D FLORET trajectory can be utilized to study lung deflation during forced expiration with high temporal resolution of 200 ms and isotropic spatial resolution of 5 mm using a TV‐regularized primal‐dual SENSE reconstruction. Acquisitions were performed for discrete distinct time frames, that is, no segmented k‐space filling across several respiratory cycles was used.

## 1 Introduction

Conventional spirometry is the clinical standard for assessing pulmonary function, particularly for characterizing obstructive and restrictive ventilatory disorders [1, 2]. Although it provides robust global metrics such as the forced expiratory volume in 1 second (FEV_1_), forced vital capacity (FVC), and the FEV_1_/FVC ratio, a fundamental limitation persists: these measurements reflect only the combined output of both lungs without any regional differentiation [3, 4, 5, 6]. Because many pulmonary diseases exhibit spatially heterogeneous early manifestations, pathological changes confined to individual segments or lobes may remain undetected [5]. As a result, diagnostic sensitivity is limited, and regional functional assessment is not feasible with standard spirometry [3, 7].

Magnetic resonance imaging (MRI) has the potential to address these limitations by enabling spatially resolved, image‐based assessment of lung function during dynamic breathing maneuvers without the usage of ionizing radiation [3, 8, 9, 10]. Previous studies demonstrated that both hyperpolarized‐gas imaging and proton‐based 3D sequences can capture the forced expiratory maneuver with high temporal resolution [3, 8]. Hyperpolarized ^129^Xe approaches have shown that spirometry‐like curves can be derived from time‐resolved signal loss during expiration, allowing the generation of regional FEV_1_ and FVC maps [3]. Parallel advances in dynamic 3D radial imaging and compressed sensing reconstruction have further shown that volumetric lung images with sub‐200 ms temporal resolution can be obtained, enabling detailed characterization of expiratory mechanics [8].

Together, these developments indicate that MRI is, in principle, capable of extending functional diagnostics by providing regional, imaging‐based spirometry. Nevertheless, methodological challenges remain, including signal‐to‐noise limitations, motion sensitivity, dependence on inhaled contrast agents, and discrepancies between image‐derived and conventional spirometric parameters [3, 8]. Additionally, the described approaches used anisotropic resolutions with reduced characterization of the coronal direction [3, 8]. Consequently, there is a need for robust MRI approaches that can capture the forced expiratory maneuver without exogenous contrast agents, with full lung coverage and high temporal resolution, thereby enabling accurate regional functional analysis.

In this work we present a 3D UTE Fermat looped, orthogonally encoded trajectory (FLORET) [11] MRI sequence that allows capturing a forced expiration maneuver and the associated rapid lung volume and motion changes with a temporal footprint of 200 ms and isotropic resolution of 5 mm allowing for image‐derived volumetry. The feasibility will be demonstrated on a custom‐built dedicated phantom and by in vivo studies.

## 2 Methods

### 2.1 MRI

Phantom data and data for volunteers 1–4 were acquired on a 3 T MAGNETOM Prisma fit scanner (Siemens Healthineers, Erlangen, Germany). Data for volunteer 5 were acquired on a 3 T MAGNETOM Skyra scanner (Siemens Healthineers, Erlangen, Germany). The body and spine coil arrays were used in each acquisition, resulting in a total of 20 receive channels. In vivo data were collected in the supine position.

### 2.2 FLORET Sequence

A custom‐built 3D FLORET [11] UTE sequence (3 hubs, cone angle 36°) implemented in Pulseq [12] was used for all measurements (TR = 1.82 ms, TE = 0.045 ms, ADC duration = 1.28 ms, *α* = 1°) with an isotropic FOV of 350 mm and an isotropic resolution of 5 mm. Sequence parameters were experimentally optimized using a custom‐built static lung phantom that models the magnetic properties of lung parenchyma as well as the custom‐built forced expiration phantom. Although a larger flip angle closer to the Ernst angle would maximize the steady‐state signal, a lower flip angle of 1° was chosen. This reduced structural background contrast, which empirically enhanced the sparsity of the data and improved the performance of the total variation (TV) reconstruction, thereby facilitating more robust image segmentation. Additionally, readout durations were kept short to mitigate off‐resonance artifacts, and the multi‐hub approach was used to balance sampling efficiency with the long‐term objective of preserving parenchymal signal definition. A fully sampled k‐space consisted of 1869 spirals (∼3.4 s) and the acquisition of it was repeated 10 times (back‐to‐back) to capture the forced expiration maneuver safely. Fast spoiling [13, 14] and a Fibonacci reordering scheme [13] using Fibonacci number 89 were applied. The trajectory was designed such that the total spoiling moment per spiral combining the native dephasing effect of the readout gradient and an explicit spoiler gradient amount to 2π. The Fibonacci number was selected based on empirical optimization, which involved evaluating undersampling and eddy‐current artifacts in phantom data. For the resulting trajectory ordering, seven spirals are acquired for a single hub before switching to the next hub, which yields a total of 21 spirals per interleave. The measurements were ungated, relying on a single continuous forced expiration without averaging or binning across cycles.

Prior to reconstruction, the nominal k‐space trajectory was corrected for system‐dependent gradient delays by applying a constant anisotropic delay (*x*: +1.0 μs, *y*: +1.5 μs, *z*: −3.5 μs). The delays were determined empirically by visually inspecting phantom and in vivo reconstructions and selecting the delay values that minimized artifacts caused by temporally shifted gradient waveforms. For reconstruction, this acquisition was divided into undersampled frames with a temporal footprint (total time span of acquired k‐space data that contributes to the reconstruction of a single image frame) of 200 ms, each consisting of 109 spiral read‐outs (undersampling factor *R* = 17).

### 2.3 Static Lung Phantom

A static lung phantom was constructed from two different types of sponges featuring distinct pore sizes and absorbencies. To mimic properties of parenchymal lung tissue, the sponges were treated with a solution of NaCl (conductivity), CuSO_4_·5 H_2_O (fungicide), and MnCl_2_·5 H_2_O (T_2_*), and distinct proton densities were adjusted (0.18 and 0.09 g·mL^−1^). Mean T_2_* times of 0.91 and 1.04 ms (3 T) were obtained for the respective sponges.

### 2.4 Forced Expiration Phantom

A custom‐built forced expiration phantom (Figure 1, total costs approximately 80 €) was constructed to further optimize and evaluate the FLORET sequence as well as the reconstruction pipeline for the proposed application. The phantom was constructed by solvent welding an acrylic glass container using methylene chloride and sealing the welded joints additionally with cyanoacrylate. The container was filled with an aqueous solution of NaCl (to adjust conductivity), CuSO_4_·5 H_2_O (as algicide), and MnCl_2_·4 H_2_O (to adjust the T_2_ relaxation time). This way, a T_2_ relaxation time of approximately 23 ms was obtained, which is comparable to the T_2_ relaxation time reported for paravertebral muscle (29 ± 4 ms) [15]. Inside the container, two breathing bags made from polyethylene terephthalate were installed—one initially filled with air and the other empty. The bags were connected through a pneumatic–hydraulic system, allowing controlled volume exchange between them using a syringe. The tube was 7 m in length with a diameter of 4 mm. This setup simulated the dynamic deflation of the lungs during a forced expiratory maneuver while maintaining a constant overall fluid volume and realistic MR signal behavior. The change of the syringe volume was monitored by a camera and the volume change was extracted and matched to the FLORET sequence by the audio track using Kdenlive (version 23.08.4) software. The dead time associated with air transport from the syringe through the tube to the breathing bag was estimated by connecting two syringes to a 7 m long tube and measuring the time delay between a volume change applied to the first syringe and the response of the second syringe. This was done again by monitoring the syringes with a camera and extracting the time delay with Kdenlive software. A mean dead time of 0.50 s was determined and used for correcting the temporal syringe volume.

**FIGURE 1:**
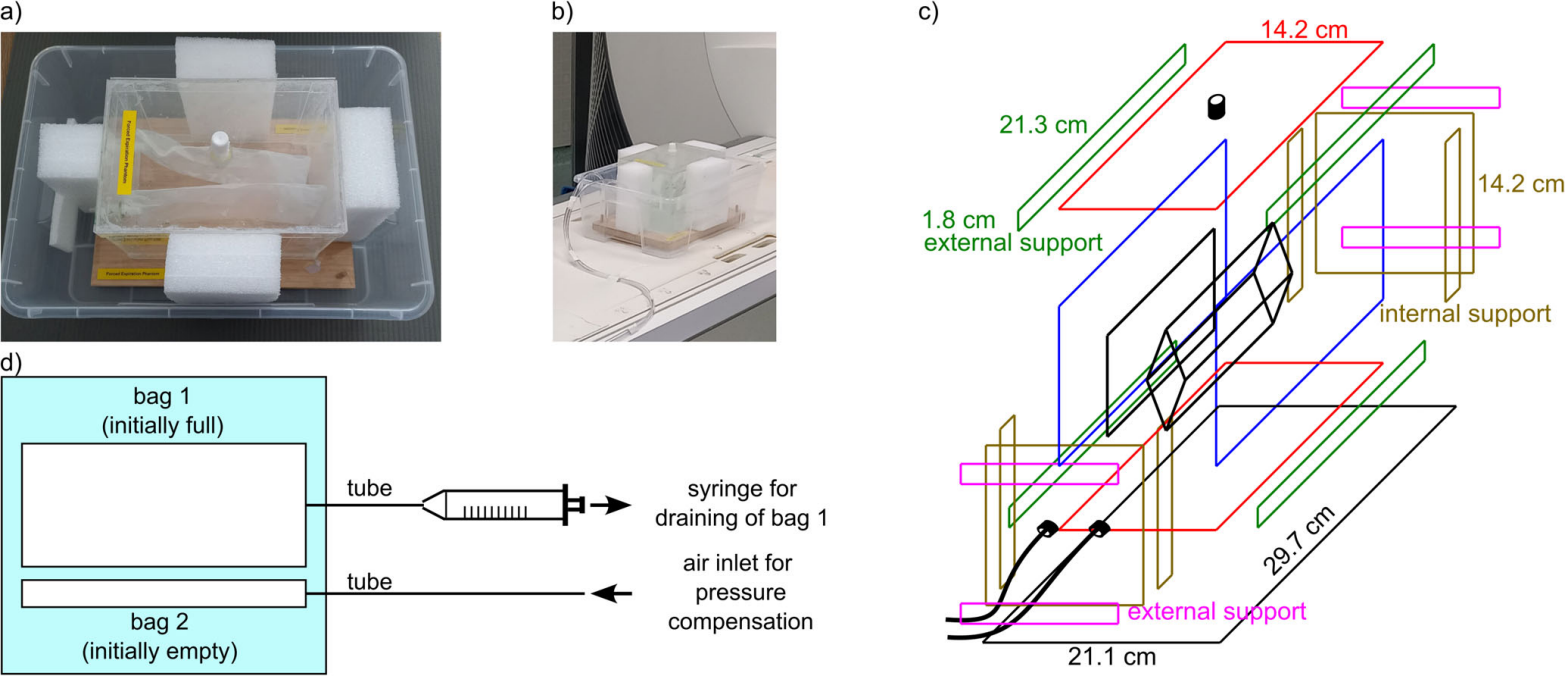
The constructed forced expiration phantom (a, b) together with the exploded view drawing (c) and a schematic drawing of the mode of operation of the phantom (d).

### 2.5 In Vivo Studies

For translation to in vivo measurements, healthy volunteers were instructed to independently inhale to maximum inspiration and hold this state for 5 s (“static front”), perform the forced expiration maneuver (“dynamic phase”), and then hold the state of maximum expiration (“static end”) for 5 s. The study was approved by the local ethics committees (ID 204/23‐sc and EA2/010/24), and written informed consent was obtained. The volunteers presented the following demographics: volunteer 1 (f/24/165 cm/57 kg), volunteer 2 (m/24/175 cm/61 kg), volunteer 3 (f/23/168 cm/69 kg), volunteer 4 (f/25/173 cm/67 kg), volunteer 5 (m/41/200 cm/89 kg).

Volunteers 1–4 were imaged at site A (Prisma), while volunteer 5 was scanned at site B (Skyra) to demonstrate multi‐site portability. For volunteer 5, a dataset of baseline upright spirometry was available.

### 2.6 Reconstruction

The forced expiration maneuver was tracked in the acquired data using a DC analysis. For this, the first point in k‐space of each spiral and coil was used, and the navigator signal was obtained by singular value decomposition and subsequent wavelet‐based smoothing as well as the application of a moving average. This way, the static front, dynamic phase, and static end could be identified.

As the body coil did not move for the phantom measurements, the coil sensitivities were assumed to be constant and the sensitivity map for the static front was used. For reconstructing in vivo data, however, sensitivity maps were modeled as time‐dependent to account for the movement of the body coil by estimating sensitivity maps for the static front and end of the acquisition and interpolating between them using the DC signal for determining the weights for the two sensitivity maps. To do so, the normalized DC signal was divided into 200 ms intervals, and the mean value of the DC signal $\epsilon_{i}$ was calculated for each interval i. Assuming the sensitivity map of the front static region is $S_{\text{front}}$ and that of the rear region is $S_{\mathrm{end}}$, then the interpolated sensitivity map $S_{i}$ in interval i was determined as

$$S_{i}=\epsilon_{i}S_{\text{front}}+\left(1-\epsilon_{i}\right)S_{\mathrm{end}}.$$

For reconstruction, the dynamic part of the acquisition was divided into undersampled frames with a temporal footprint of 200 ms, each consisting of 109 spiral read‐outs. These were then reconstructed using an iterative, TV‐regularized, non‐Cartesian SENSE reconstruction employing a primal‐dual optimization algorithm. Spatial (TVs) and temporal (TVt) total variations were employed.

$$\min_{x}\frac{1}{2}{\left\|\mathrm{Ex}-y\right\|}_{2}^{2}+\lambda_{t}{\left\|\nabla_{t}x\right\|}_{1}+\lambda_{s}{\left\|\nabla_{s}x\right\|}_{1}$$

Here, E is the non‐Cartesian SENSE encoding operator (including coil sensitivities determined by Joint SENSE calibration and NUFFT), y is the measured k‐space data, $\nabla_{s}$ and $\nabla_{t}$ denote the spatial and temporal gradients, respectively, and $\lambda_{s}$ and $\lambda_{t}$ are the spatial and temporal TV regularization weights, which were optimized empirically as 0.03 and 0.1, respectively. x is the complex‐valued spatiotemporal image to be reconstructed.

Reconstruction was performed on a 70^3^ matrix for the phantom and a 128^3^ matrix for in vivo data to minimize computational costs and ensure compatibility with GPU memory constraints. Subsequently, the data were zero‐filled by a factor of 2 in each dimension to achieve a final matrix of 140^3^ and 256^3^, respectively for the segmentation process.

### 2.7 Implementation

The navigator signal for DC analysis and modeling the coil sensitivities was generated from the raw data using a custom MATLAB code (MATLAB R2023b (Mathworks, Natick, MA)). The DC signal was generated from all coils using a singular value decomposition followed by wavelet denoising and a moving average. Extraction of the relevant part of the breathing maneuver and calculation of the density compensation required for the subsequent gridding procedure were also performed in MATLAB. The main constrained SENSE reconstruction was then performed employing a custom Python code. Reconstructed data were segmented in MATLAB using the built‐in Volume Segmenter app as part of the Image Processing Toolbox.

### 2.8 Example Data

An exemplary phantom data set with the used Pulseq sequence and reconstruction can be found under DOI: https://doi.org/10.5281/zenodo.20431504.

## 3 Results

High quality images could be obtained by applying the proposed reconstruction approach to the undersampled data for the phantom and in vivo data. For the phantom, the temporal progression of syringe volume, DC signal (normalized from 0 to 1 and scaled to the minimum/maximum syringe volume), and segmented volumes were compared (Figure 2a,b). Even though the deformation of the breathing bags is much more irregular and less well defined than for a lung, depictions with well‐defined object boundaries could be obtained (Figure 2b). The measured syringe volume corresponding to a FVC was 325 ± 10 mL. From the segmented data, the FVC was obtained by the difference of the segmented mean values for the first 8 and last 8 segmented volumes of the static front and end frame, respectively, as 327 mL. The standard deviations for the static front and end were 9 and 11 mL, respectively, and the standard errors of the mean were 3 and 4 mL, respectively. For the syringe, the volume change within the first second serving as an MRI‐based FEV_1_ surrogate yielded 105 mL while the segmented data yielded 100 ± 10 mL (error estimated from the segmented static regions). While the phantom's absolute volumetric dynamics were constrained by pneumatic tubing resistance to maintain a compact, cost‐effective design, the highly irregular deformation of the internal breathing bag still provided a sufficiently complex motion environment to rigorously test the acquisition and reconstruction.

**FIGURE 2:**
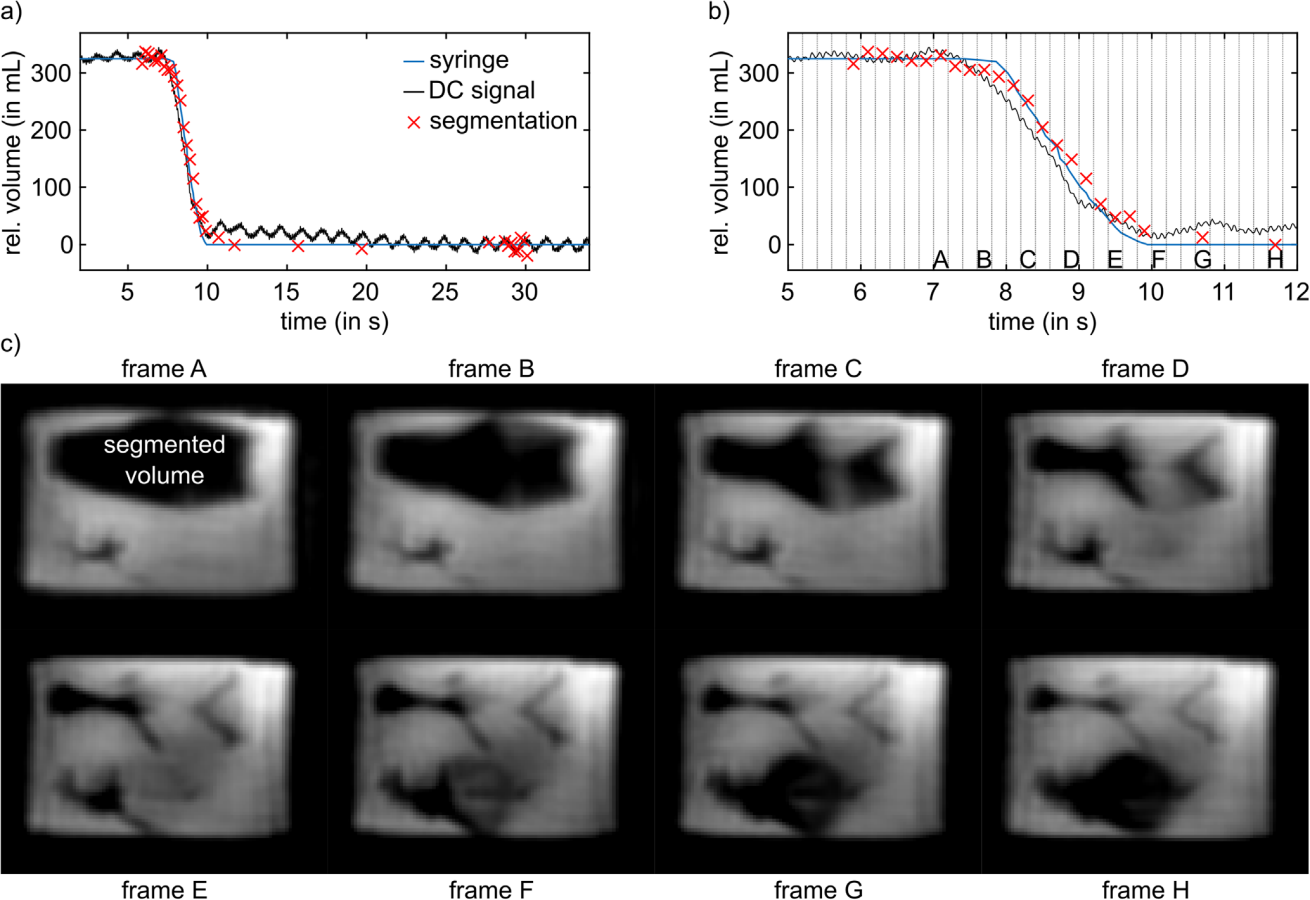
(a) Comparison of the syringe volume, DC signal (normalized from 0 to 1 and scaled to the minimum/maximum syringe volume) and segmented volumes for the “upper” bag of the forced expiration phantom in (c). (b) shows an enlarged part of (a) together with the indicated individual frames (vertical lines). Additionally, selected frames are marked with letters, for which a coronal slice is shown in (c). TR = 1.82 ms, TE = 0.045 ms, FOV = 350 mm, resolution = 5 mm.

The in vivo data were also segmented to obtain time dependent lung volumes (Figure 3b, volunteer 1). Also, for the in vivo data, images of high quality were obtained even for lung movement during forced expiration (Figure 3, volunteer 1). From the segmented volumes, several quantitative parameters were derived, including the duration of the dynamic phase (2.8 ± 0.2 s), the time to 90% deflation (1.8 ± 0.2 s), the volume change within the first second (2.8 ± 0.2 L) and the total volume change (3.5 ± 0.2 L) (the volume error was estimated from the segmented static regions, the time error was assumed to be determined by the temporal resolution). Volumetric curves were approximated by interpolating between the reconstructed data points for evaluation. The volume change within the first second serves as an MRI‐based FEV_1_ surrogate; however, it is not equivalent to the FEV_1_ value obtained from conventional spirometry, as discussed below. Similarly, the FVC and resulting FEV_1_/FVC ratio represent an MRI‐based surrogate and do not directly correspond to spirometric measurements. The MRI‐based FEV_1_/FVC ratio surrogate was determined as 0.79.

**FIGURE 3:**
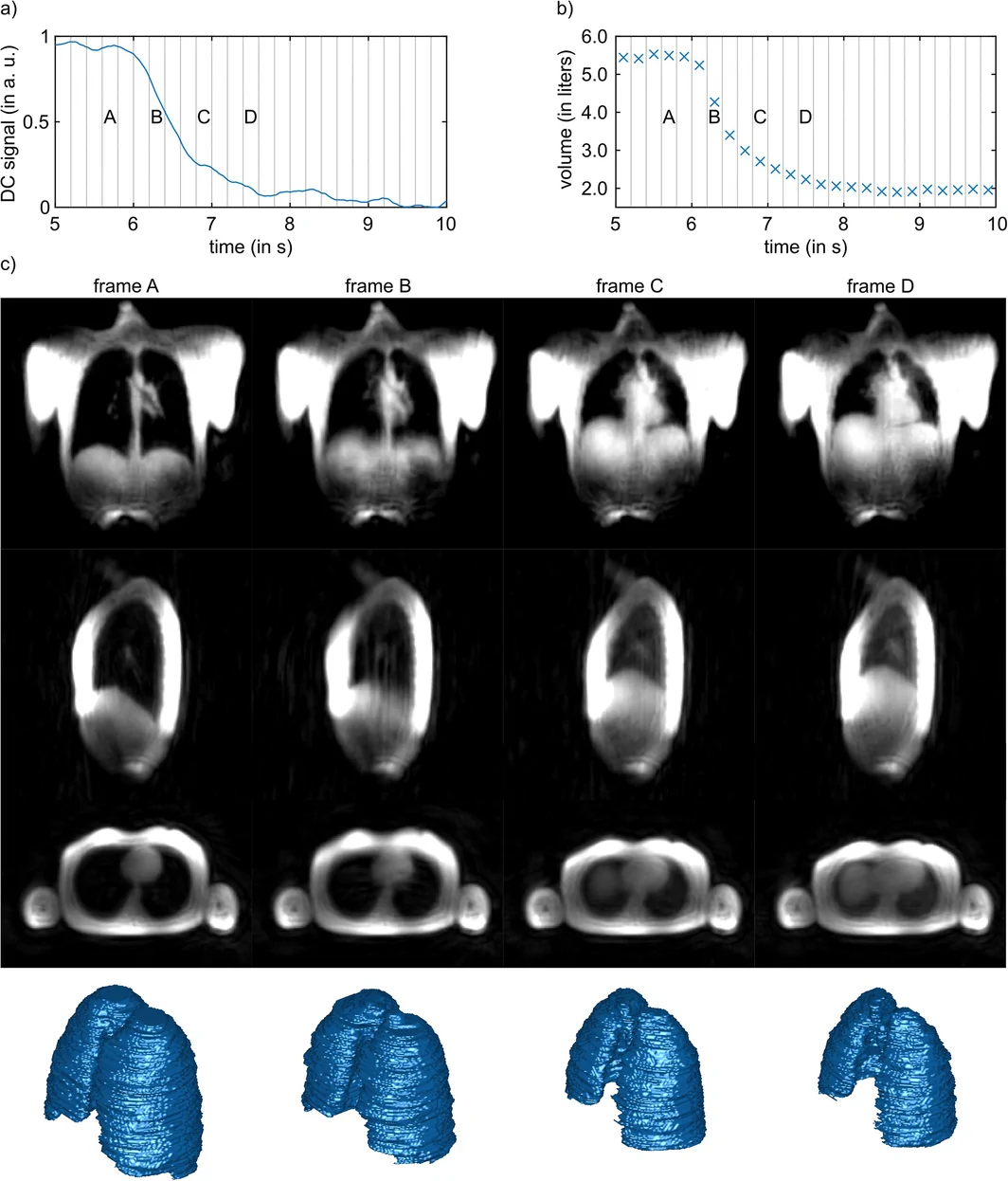
(a) DC signal and (b) segmented volumes for the in vivo measurement of volunteer 1 with indication of the individual frames (vertical lines) and selection of four frames (letters) for which a coronal, sagittal and axial plane as well as the segmented lungs are shown in (c). TR = 1.82 ms, TE = 0.045 ms, FOV = 350 mm, resolution = 5 mm.

Additionally, the segmentation of the in vivo data allowed to discriminate between the left and right lung lobe (Figure 4a, volunteer 1). For the right and left lung lobe, the total volume changes were determined as 1.8 ± 0.2 L and 1.7 ± 0.2 L, the volume change within the first second as 1.8 ± 0.2 L and 1.7 ± 0.2 L, and the MRI‐based FEV_1_/FVC ratio surrogates as 0.83 and 0.78. MRI‐derived peak expiratory flow (PEF) values (ranging from 280 to 400 L·min^−1^ depending on the fitting evaluation) were lower than standard clinical values obtained from upright spirometry (∼500 L·min^−1^).

**FIGURE 4:**
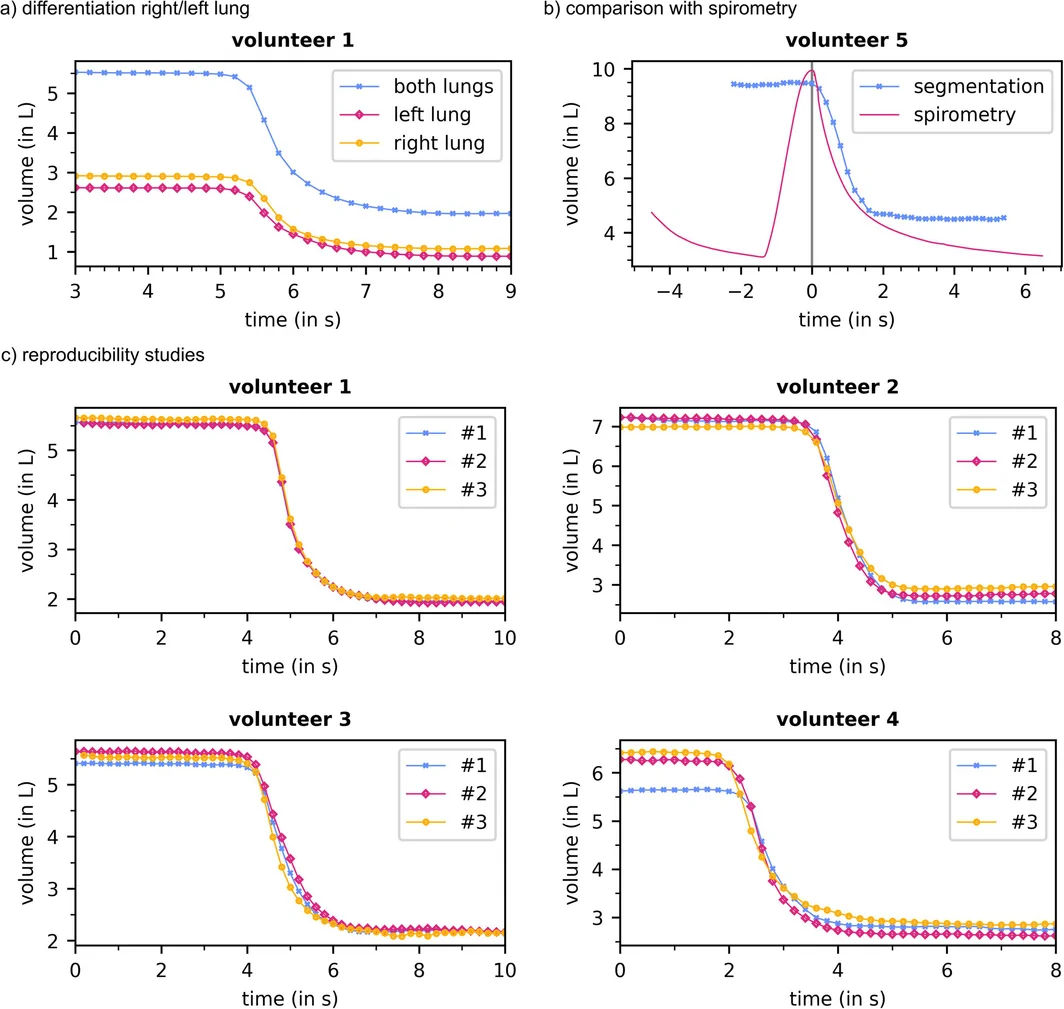
(a) Segmented volumes for one volunteer for the whole lung, left lung and right lung. (b) Comparison of the segmented lung volumes (supine position) and the results from conventional spirometry (sitting position) for a healthy volunteer. (c) Reproducibility studies of the forced expiration MRI protocol for four healthy volunteers. Each volunteer repeated the breathing maneuver in three individual measurements.

Comparison of MRI‐derived segmented volumes and conventional spirometry for volunteer 5 (Figure 4b) showed substantial differences in the volumetric data.

For reproducibility evaluation, volunteers 1–4 underwent three individual measurements each (Figure 4c). FVC surrogates were: 3.6, 3.6, and 3.6 L for volunteer 1; 4.6, 4.5, and 4.0 L for volunteer 2; 3.3, 3.5, and 3.4 L for volunteer 3; 2.9, 3.7, and 3.6 L for volunteer 4. FEV_1_ surrogates were determined as: 2.5, 2.5, and 2.5 L for volunteer 1; 3.4, 3.1, and 2.6 L for volunteer 2; 2.1, 2.0, and 2.1 L for volunteer 3; 2.1, 2.4, and 2.4 L for volunteer 4. FEV_1_/FVC surrogates yielded: 0.69, 0.69, and 0.69 for volunteer 1; 0.74, 0.69, and 0.65 for volunteer 2; 0.64, 0.57, and 0.62 for volunteer 3; 0.72, 0.65, and 0.67 for volunteer 4.

For the in vivo measurement, data with and without TVs and TVt were compared (Figure 5), showing how the TV aids in recovering image quality for highly undersampled data.

**FIGURE 5:**
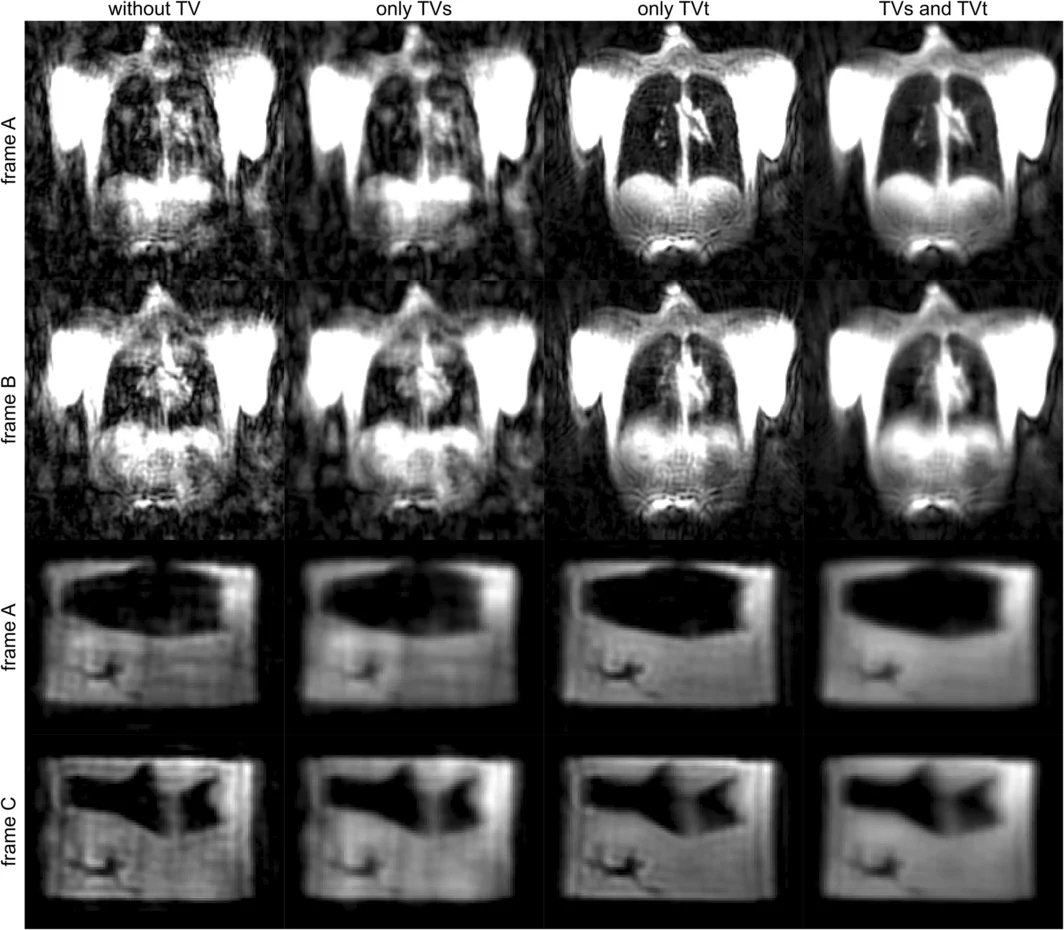
Comparison of the influence of the TV regularization for two frames of volunteer 1 and the phantom. From left to right, no regularization was used, only TVs, only TVt and both regularizations.

## 4 Discussion

Our study demonstrated the feasibility of using an undersampled 3D‐FLORET trajectory and a model‐based reconstruction method to investigate forced expiration with high precision. Both the acquisition in phantom and a healthy volunteer indicate the high fidelity of the spatio‐temporal depictions in “real‐time,” despite fast object movement.

The temporal course of the syringe volume (Figure 2a,b) was corrected with a temporal shift (dead time) which is caused by the resistance of the long, thin tube and the turbulent flow. The determined FVC values were almost identical for the syringe measure and the segmented volumes, proving that despite the high undersampling, the static regions can be reconstructed accurately. The standard deviation and standard error of the mean were larger for the static end, which is due to the highly irregular shape of the deflated breathing bag and residual movement. The temporal course of the syringe volume deviates from the temporal course of the segmented volumes, as a simple linear delay correction is not sufficient for the data because the system operates at high Reynolds numbers and therefore in the regime of turbulent gas flow. In addition to the pure transport delay of the air and the compressibility of the air within the tubing, this leads to a nonlinear response of the breathing bag to changes in the syringe volume. This is evident from the increasing lag of the segmented data behind the syringe volume over time. The slightly faster volume loss of the segmented data compared to the syringe volumes at the beginning of the dynamic phase is not due to a nonlinear response, but rather to a rapid deformation of the breathing bag caused by water pressure when syringe operation commences. Nevertheless, the determined FEV_1_ surrogates are in good agreement, indicating that precise segmentation is achievable even for the dynamic frames.

Regarding all determined volume errors, it is critical to note, however, that these were estimated based on the segmented static images. While this provides a robust measure of the static segmentation uncertainty, it underestimates the true volumetric error as it neglects additional factors such as motion and blurring. Consequently, the reported error should be strictly interpreted as the baseline segmentation error, whereas the total operational error during the real‐time breathing maneuvers is expected to be higher.

While the movement of a lung is quite restricted, the PET bags are deformed much more strongly and much more irregularly (Figure 2c). In addition to asymmetrical rapid folding, the buoyancy of the bags changes, resulting in additional movement in the coronal direction. The displacement of the solution also causes additional movement. Nevertheless, high‐quality images are obtained and the segmented relative volumes follow the DC signal closely. This infers that the reconstruction yields meaningful results even for rapid (and asymmetric) movements, thus validating the feasibility of the method. No temporal smoothing due to excessive TV weighting could be detected, which could happen as discussed in the literature [16, 17].

Thus, the phantom data motivate that the method can be transferred to in vivo measurements. For the in vivo data, even though segmentation of the lung is possible (Figure 3c), there is no ground truth the segmented values can be compared to like conventional spirometry due to various reasons: (i) Clinical spirometry and MRI based segmentation measure fundamentally different values, as extensively discussed by Berman et al. [8] A spirometer measures only the volume of gas expired through the mouth and therefore reflects the total gas volume displaced from both lungs during expiration. In contrast, image‐based segmentation quantifies the loss of lung voxels between consecutive frames and thus captures not only changes in gas volume but also contributions from lung tissue volume [8]. While the solid cellular components of the lung parenchyma do not compress significantly during respiration, the lung volume segmented on macro‐scale MRI encompasses both these cellular structures and the intravascular pulmonary blood volume [18, 19]. Because the lung acts as a highly compliant vascular reservoir, the substantial intrathoracic pressure changes generated during a forced expiration maneuver can displace several hundred milliliters of blood out of the thoracic cavity, reducing the pulmonary blood volume by up to 50% [18]. Consequently, the dynamic volume changes captured by our MRI framework reflect a combination of gas displacement and this rapid hemodynamic redistribution [18, 19]. (ii) The spirometric metrics differ if spirometry is performed in the sitting (clinical spirometry) or supine position (MRI), as shown by Bewes et al. [3] (iii) The breathing maneuver with the static front differs from the clinical spirometry, which may lead to inconsistencies (Figure 4b).

MRI‐derived peak expiratory flow (PEF) values (ranging from 280 to 400 L·min^−1^ depending on the fitting evaluation) were lower than standard clinical values. The deviation of the MRI‐derived PEF from standard clinical values is driven by the well‐documented physiological reduction in PEF associated with the supine imaging posture [3], combined with the achieved temporal resolution.

Even though the forced expiration maneuver is rapid, high‐quality images for the lung were obtained Figure 3c. However, especially for the highly dynamic frame B, artifacts are present, like blurring of the diaphragm boundary. Nevertheless, the contours of the lung can be robustly identified for every frame, which is essential for reproducible segmentation. This is mirrored in the segmented volumes in Figure 3b, which result in a smooth expiration process.

The test–retest evaluation demonstrates a high overall reproducibility of the MRI‐derived spirometric surrogates, proving the technical stability of the underlying reconstruction and automated segmentation pipeline. For instance, volunteer 1 exhibited identical metrics across all three consecutive runs, while volunteer 3 showed only marginal intra‐subject variance. Crucially, because these image‐derived metrics reflect active physiological execution, their reproducibility is inherently linked to subject compliance and maneuver consistency. This dependency is distinctly visible in the first run of volunteer 4; an incomplete initial inspiration led to visibly reduced surrogates (FVC: 2.9 L, FEV_1_: 2.1 L) compared to the highly stable and successful subsequent maneuvers (FVC: 3.7 L and 3.6 L; FVC: 2.4 L). Similarly, slight progressive variations observed in volunteer 2 likely capture minor physical fatigue or altering effort between repetitions; a phenomenon well‐documented even in standard clinical spirometry [20, 21]. Overall, the data confirm that when proper maneuver compliance is maintained, the proposed framework yields robust and reproducible functional biomarkers.

Comparing the data with and without TVs and TVt in Figure 5 reveals that even in the absence of any TV the lung contours are defined. This is due to the highly isotropic sampling pattern of the FLORET trajectory and the applied trajectory ordering scheme with Fibonacci number 89. The TVt leads to major improvement in the image quality, whereas the TVs also introduce slight blurring, but were beneficial in the segmentation of the phantom data.

Segmentation of the lung volume allowed the two lung lobes to be analyzed separately with respect to MRI‐based surrogates of pulmonary metrics. This differentiation provides an initial step toward identifying regional variations in lung behavior during forced expiration.

Compared to the work by Berman et al. [8], the use of a 3D FLORET trajectory for the forced expiration maneuver yielded usable image quality even in the absence of TV regularization. This indicates an intrinsically improved data consistency and sampling efficiency of the proposed acquisition scheme. Furthermore, no additional “bookend” TV regularization incorporating the static pre and postexpiratory states was applied, thereby avoiding potential bias toward these reference states. This reduced reconstruction dependencies on strong prior assumptions suggests a more robust encoding of the dynamic expiration process. The good agreement between the segmented volumes and the syringe volumes in the phantom experiments also suggests that no significant temporal smoothing occurs. The isotropic resolution of the FLORET trajectory allowed for an isotropic resolution of 5 mm compared to the anisotropic resolution of 4.6 × 4.6 × 10 mm by Berman et al. [8] Additionally, the acceleration factor was able to be decreased from 38 for a temporal footprint of 208 ms [8] to 17 for a footprint of 200 ms.

The presented findings clearly demonstrate that the 3D FLORET trajectory has high potential and is advantageous for the investigation of rapid lung movement during a forced expiration maneuver and for MRI based spirometry. However, there are still limitations and future improvement is necessary to facilitate the feasibility for clinical translation. As the segmented lung volumes do not correspond to classical volumes determined by spirometry, values determined from image‐derived volumetry will have to be interpreted as separate biomarker and not as an identical substitute for conventional spirometry. A more conclusive evaluation will require the inclusion of patient data, which would allow the identification and analysis of potential regional anomalies. Furthermore, the absolute volume change and the flow rates of the forced expiration phantom were not fully matched to adult in vivo expectations.

## 5 Conclusion

The phantom and in vivo studies demonstrate, that the 3D FLORET trajectory can be utilized to study lung deflation during forced expiration with high temporal resolution of 200 ms and isotropic spatial resolution of 5 mm using a TV‐regularized primal‐dual SENSE reconstruction. Acquisitions were performed “real‐time,” that is, no segmented k‐space filling across several respiratory cycles was used and high‐quality image data were obtained.

## Funding

This work was funded by the German Research Foundation (project number 535829883).

## Conflicts of Interest

The authors declare no conflicts of interest.

## Data Availability

An exemplary phantom data set with the used Pulseq sequence and reconstruction can be found under DOI: https://doi.org/10.5281/zenodo.20431504. Further data that support the findings of this study are available from the corresponding author upon reasonable request.
